# Life in a London Hospital. I

**Published:** 1890-07-26

**Authors:** 


### LIFE IN A LONDON HOSPITAL.—I.

SOME months ago it was my privilege to become a patient in a surgical ward of one of the largest hospitals in London. Whilst there I had ample opportunity of observing everybody and everything, so it may perhaps interest my readers to hear a little about the kind of life that is led there. There were twenty-eight beds in the ward, fourteen on each side, placed at a good distance one from another, so that anything like overcrowding was avoided. Over each bed was a printed number, for there all the patients were called and spoken of by their own particular number. Indeed, after I had been in the ward a few days, I began to lose my own identity altogether, and could only realize that I was No. 28—one among many other sufferers. There was also a picture over each bed, and on the right hand side of the patient, between the window and next bed, stood what is called a "locker," where we kept all our belongings; this locker also served as a seat for our visitors, who would take it in turns to sit down every ten minutes or so during the visiting hours. There were large tables placed along the centre of the ward, on which were tastefully arranged plants and flowers, brought by kind friends from the outside world.

Two or three arm chairs, a large comfortable sofa, and several reclining chairs were grouped about close to the fire, forming almost a little sitting room for the patients who were well enough to be out of bed, and there was also a small harmonium used when the hymns were sung at the close of day.

We breakfasted at five every morning, and as soon as that meal was over the proceedings of the day began in earnest. Talking was not allowed till six o'clock, but immediately Big Ben chimed forth that hour a chorus of voices began. "Good-morning No. 6, good-morning No. 2, good-morning No. 8," and so on, until all special friends had duly exchanged greetings, and then, whilst the ward was being swept and the patients were washed, there was a continual flow of conversation touching on all topics, and I often noticed that there was a great amount of sympathy expressed by every one for the poor patient who was to undergo an operation during the course of the day. Even the children in the ward seemed to look upon an operation as quite a natural and necessary occurrence.

One little girl assured me very soon after my arrival at the hospital, that after I had been there a week I was sure to have an operation. Well, she was quite right, and so I did, but it astonished me very much to hear the calm, matter-of-fact way in which she pronounced my sentence.

At eight the "Sister" of the ward read prayers, and by nine all was tidy and we were ready for our lunch of bread and milk. Jam was allowed to those who had it brought them by their friends, and I remember a dear little girl leaving her bread and milk in my charge whilst she went to fetch some jam out of her locker. It was amusing to see the abundant way in which she spread the jam on her bread, at the same time holding out the pot for me to have some too. She had evidently no thought for the morrow or its needs.

This small friend of mine was called "Jenny." She was a pretty child of six, with deep blue eyes shaded by the loveliest eyelashes, and her dark hair was cut short like a boy's. She was quite a little character in her way. When we had finished lunch the ward was once more swept and everything put away so that we should be ready for the house-surgeons, who made their rounds from ten till twelve. There was a house-surgeon to each side of the ward, and each of them was generally accompanied by a dozen or so of students.

In No. 24 bed there was a little girl named "Alice," suffering from a very bad form of hip-disease. She was a tiny, shrivelled-up child of seven, and her poor little legs were so thin that they actually seemed to be smaller than my wrists. She used to cry dreadfully when the surgeon was dressing her wounds; it was pitiful to hear her; even now her voice rings in my ears as she cried out then, "Oh! it hurts, oh! it does hurt, it does." I do not think I shall ever forget the piteous little cry, it used to haunt me all the day afterwards.

After I had left the hospital I went back to see my old ward; there was only "Alice" there that I remembered. I found her much better and very happy because she was going into the country, and going about in a little carriage, so she told me. One of the nurses lifted her out of bed to kiss me, and whilst I was stroking her little legs the child suddenly remarked, "One of my legs is a long leg, much longer than the other, I shall have to have a wooden leg 'cos one leg is so long, and the other is so short."

No. 3 was another young friend of mine. She had been obliged to have one of her legs amputated above the knee, but she had recovered sufficiently to be allowed up, and it was really wonderful to see how she managed to move herself about in the wheeled chair. She would often come and sit by my bed-side and entertain me with her past experiences. She told me that she had lost her leg through a kick from another child, but she seemed to bear this child no ill-will; indeed, she looked the picture of happiness and contentment. Judging by a little incident the "Sister" of the ward told me concerning her, I think she must have been of an unusually sweet and unselfish disposition. It appears that when she recovered from the effects of the ether after her leg had been amputated, she began to cry, "Oh! my leg, oh! my poor leg," then in the same breath she added, "What will mother say?" showing that she thought more of her mother than of herself. Her bed was almost opposite mine, and it was curious to note how perfectly at home she seemed with all the doctors and students. I saw them all laughing heartily one morning at some remark she had just made to them.

There was one student who I shall always think of with kindness. He had such a bright cheerful face, and a most winning manner of speaking. I need not say that all the students were kindness itself, whenever they had anything to do for us, but this particular one I seem to remember the best. As he passed down the ward he would always stay a minute or so to ask me how I felt, and to make some remark of no great importance, perhaps, to other people, but it used to please me. One day he asked me if I had many letters from home? and another time he said he hoped I intended to try and eat some dinner. Very common-place remarks, but nevertheless, I have not forgotten the kind voice in which they were uttered.

E. M. C.

*(To be continued.)*

